# Hidden Epistastic Interactions Can Favour the Evolution of Sex and Recombination

**DOI:** 10.1371/journal.pone.0048382

**Published:** 2012-11-21

**Authors:** Joel R. Peck, David Waxman, John J. Welch

**Affiliations:** 1 Department of Genetics, University of Cambridge, Cambridge, United Kingdom; 2 Centre for Computational Systems Biology, Fudan University, Shanghai, PRC; 3 Department of Genetics, University of Cambridge, Cambridge, United Kingdom; British Columbia Centre for Excellence in HIV/AIDS, Canada

## Abstract

Deleterious mutations can have a strong influence on the outcome of evolution. The nature of this influence depends on how mutations combine together to affect fitness. “Negative epistasis” occurs when a new deleterious mutation causes the greatest loss in fitness in a genome that already contains many deleterious mutations. Negative epistasis is a key ingredient for some of the leading hypotheses regarding the evolution of recombination, the evolution of sex, and a variety of other phenomena. In general, laboratory studies have not supported the idea that negative epistasis is ubiquitous, and this has led to doubts about its importance in biological evolution. Here, we show that these experimental results may be misleading, because negative epistasis can produce evolutionary advantages for sex and recombination while simultaneously being almost impossible to detect using current experimental methods. Under asexual reproduction, such hidden epistasis influences evolutionary outcomes only if the fittest individuals are present in substantial numbers, while also forming a very small proportion of the population as a whole. This implies that our results for asexuals will apply only for very large populations, and also limits the extent of the fitness benefits that hidden epistasis can provide. Despite these caveats, our results show that the fitness consequences of sex and recombination cannot always be inferred from observable epistasis alone.

## Introduction

For several decades, experimentalists have been trying to understand the way that deleterious mutations combine to affect fitness. Much attention has been paid to “negative epistasis” among deleterious mutations, which occurs when a new deleterious mutation causes the greatest loss in fitness in a genome that already contains many deleterious mutations. If negative epistasis is common, then it can help to resolve a variety of difficult questions in evolutionary biology [1]–[10]. These questions include well-known conundrums such as why obligate sexual reproduction has evolved in many species, despite the large associated costs. Another problem that can be addressed by negative epistasis involves drift load. Drift load refers to the decrease in fitness that occurs when small-effect deleterious mutations rise to high frequencies as a result of genetic drift. Drift load can have a very large negative impact on mean fitness, particularly when the number of sites under selection within the genome is large in comparison to local population size. Without negative epistasis, it is difficult to understand how many eukaryotic populations can survive, particularly in the case of organisms that typically have relatively small local population sizes, like most trees and large mammals [7]. In addition, if drift-load levels are high, then small changes in population size should have large effects on mean fitness, and this is not generally observed [7], [11]. Negative epistasis can ameliorate this situation, reducing both drift load and the sensitivity of fitness to population size [7], [11].

Because of its potential importance, negative epistasis has been a focus of experimental work, and a variety of approaches have been adopted to test for it in natural and laboratory settings, including direct comparisons of single and double mutant fitnesses, observations of the decline in fitness in mutation accumulation lines, and crosses of fit and unfit individuals [12]–[16]. The findings of such experiments have not generally supported the prevalence of negative epistasis. Indeed studies on a variety of microorganisms show evidence of positive epistasis (where deleterious mutations are most harmful in the least mutationally contaminated genomes). These findings have been used to question the importance of negative epistasis in evolution [13], [17], [18]. Here, we consider biologically-motivated fitness landscapes that differ from those considered in most previous studies. We demonstrate that some fitness landscapes can produce an advantage for sex and recombination even when experiments of the sort that are currently performed would be unlikely to find any trace of negative epistasis. Indeed, sex and recombination can be favoured even when experiments are likely to suggest that positive epistasis is common.

## Results

### Model

We will consider variants of a simple model that has been used by a number of previous authors to study the evolution of sex and recombination [5], [6], [19]–[21]. We consider a population of effectively infinite size (such that random genetic drift can be neglected) with discrete non-overlapping generations. The genome is diploid, and contains a very large number of sites that are susceptible to deleterious mutations. Mutations occur independently of one another, and at the same rate at all sites. As such, the distribution of new mutations per individual per generation is Poisson with rate 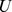.

We assume that selection takes place only on the diploid phase of the lifecycle, but given our assumptions about reproduction (see below), fitness can be taken to include the effects of viability (survival to reproductive age) and fertility. We will assume that the relative fitness of an individual carrying 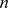 deleterious mutations is given by:
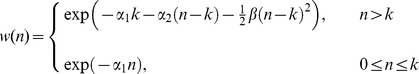
(1)Since we never consider beneficial mutations, fitness cannot increase with the number of mutations carried. We therefore assume that 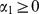, 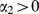, 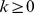 and further, when 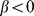 and 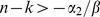 we take 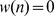 [19]. Following previous authors [5], [19]–[21], our fitness function assumes that there is no dominance, i.e., we assume multiplicative interactions within loci. However, this assumption has no effect on our results, because population size and number of loci are assumed to be large enough for homozygotes to be vanishingly rare [19]. Below, we will consider results that are special cases of this generalised fitness function, and indeed, Eq. (1) is sufficiently flexible to contain those of several previous authors as special cases [5], [6], [19], [20], [21].

We consider three different modes of reproduction. The first of these is asexual reproduction, which involves the production of offspring that are, apart from any new mutations, genetically identical to their mothers. The second is sexual reproduction with free recombination. Here, the population is hermaphroditic, and undergoes random union of gametes and free recombination among sites. Finally, we consider sexual reproduction with no recombination. This also assumes an hermaphroditic population and random union of gametes, but in this case the genome consists of two non-recombining haplotypes, and, apart from any new mutations, each gamete consists of a randomly selected copy of one of the two haplotypes of a parental adult. We will call these modes of reproduction asexuality, free recombination, and segregation only, respectively. The full population genetic recursions under these three modes of reproduction are given below (Eqs. (11), (13) and (14)).

Following previous studies, [5], [6], [19], [21], [22], we will focus below on how the different modes of reproduction affect mean fitness at mutation-selection equilibrium, a quantity denoted 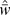. We will also describe the equilibrium populations in terms of the mean number of mutations carried, 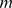, and the variance in the number of mutations carried, 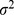. Full derivations of all results presented can be found in the Analysis section.

### Results without hidden epistasis

**Figure 1:**
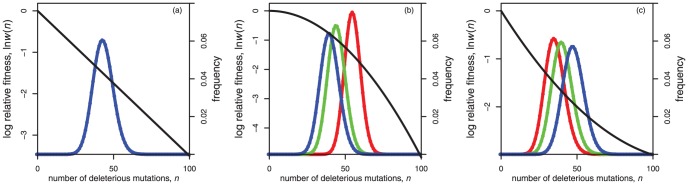
Fitness landscapes and equilibrium frequency distributions. Each plot has two vertical axes. The left-hand-vertical axis gives the natural logarithm of relative fitness, and it relates to the black lines that appear in each plot. The right-hand vertical axis gives the relative frequency of the various genotypic classes, and it relates to coloured lines and points. The three colours give results for asexuality (red), free recombination (blue), and segregation only (green). All three coloured lines occur in exactly the same positions in panel (a) because this plot shows the data when epistasis is absent, in which case all three modes of reproduction lead to the same distribution of genotypic classes. Panel (b) shows a case of negative epistasis, which leads to a disadvantage for asexuality. Panel (c) shows a case of positive epistasis, which leads to an advantage for asexuality. The fitness landscapes shown are from Eq. (1). The parameters used for the three panels were: (a) 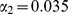, 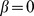; (b) 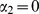, 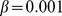; (c) 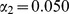, 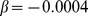. In all cases, 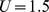, 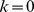.

It is now well established that when mutations affect fitness independently (i.e., when each successive mutation reduces fitness by a fixed factor, as shown in Figure 1a), the foregoing model leads to the same equilibrium mean relative fitness, regardless of the mode of reproduction. In particular, in this case, we have 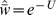. When mutations affect fitness independently, log fitness is a linear function of the number of mutations, and epistasis can be modelled by introducing curvature into this function, as shown Figures 1b and 1c [19]. When there is negative epistasis, log fitness is concave-down, as a function of the number of deleterious mutations (Figure 1b). In this case, the mean relative fitness for asexuals is still 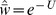 (indeed this value always holds for asexuals, so long as fitness declines towards zero as the number of deleterious mutations increases). However, negative epistasis leads to enhanced mean relative fitness for both free recombination and segregation only (both modes of reproduction lead to 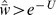). Furthermore, under negative epistasis, the equilibrium mean relative fitness for free recombination generally exceeds the equilibrium mean relative fitness for segregation only [19]. Finally, with positive epistasis (Figure 1c), equilibrium mean relative fitness is typically highest for asexuals and lowest for free recombination, with segregation only achieving an intermediate level of fitness [19]. This occurs when log fitness is concave-upwards, as a function of the number of deleterious mutations.

With our general fitness function, Eq. (1), the results shown in Figure 1 are modelled by setting (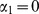 and 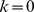). The sign and strength of the epistasis then depends on the curvature parameter 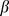, with 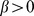 specifying negative epistasis and 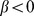 specifying positive epistasis. Charlesworth [19] presented an extensive analysis of this fitness function, and below we provide new closed-form analytical approximations for Charlesworth's model.

### Results with hidden epistasis

If real-world fitness landscapes had log-fitness functions with constant curvature, like those in Figure 1, then existing experimental approaches should be sufficient to detect epistasis. Indeed the relevant parameters (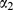 and 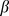 in our notation) have been estimated by experimentalists [12]–[18]. As such, the failure to consistently detect negative epistasis has been considered strong evidence against the importance of this sort of interaction in evolution [13], [17]. We show here that this rejection might be premature. In particular, negative epistasis can lead to advantages for sexual reproduction, even if log fitness is a linear function of the number of deleterious mutations for all genotypes that an experimenter is likely to observe. This can occur when the portion of the selection function that is non-linear occurs in a region of “genotype space” that is occupied only by rare individuals: those that have genomes with very low numbers of deleterious mutations (relative to the rest of the population). Negative epistasis can thus influence evolutionary outcomes despite being difficult to detect using standard experimental methods.

The simplest way to demonstrate the sort of effects we wish to describe here is to consider fitness landscapes in which fitness is unaltered by each of the first 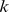 mutations that are added to an individual's genome. Each additional mutation, causes a decrease in log fitness by an amount denoted 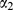 (we obtain this fitness function by setting 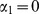 and 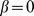 in our Eq. (1)). The resulting fitness function contains a neutral “ridge” and so, when 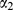 is large, it corresponds to the truncation selection model of Kondrashov [21]. The important thing to note about this landscape is that it is exhibits negative epistasis only when we compare individuals containing 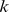 or fewer mutations with individuals containing more than 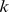 mutations. All other comparisons show no evidence of epistasis.

**Figure 2:**
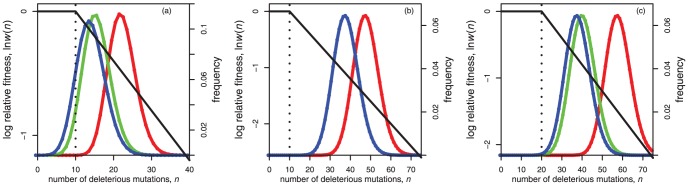
Fitness landscapes and equilibrium frequency distributions. With the fitness landscapes shown, the first few mutations have a negligible effect on fitness until they reach a threshold. After that, each mutation decreases fitness by around 5%. Parameters for the three panels were: (a) 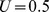, 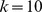; (b) 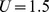, 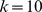; (c) 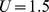, 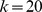. In all cases, 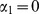, 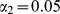, 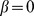. All other conventions (axes, colour-coding etc.) as Figure 1. Note that in panel (b), sexual populations with and without recombination attain the same equilibrium (such that the green curve is hidden); while in panel (c), the red (asexual) curve would be unlikely to apply to any population of plausible finite size, as drift would be likely to dominate the outcome.

Figure 2 displays the equilibrium populations under this model for some illustrative parameters. In Figure 2a, in comparison to asexuals, both segregation and recombination lead to increases in equilibrium mean relative fitness due to the negative epistasis. However, regardless of the experimental approach adopted, this epistasis would be easily detectable only with the sexual populations. For the asexuals, only a very small fraction of the population carry fewer than 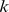 mutations at equilibrium. These rare individuals would not be present in a typical sample from a population, unless the sample size was extremely large.

**Figure 3:**
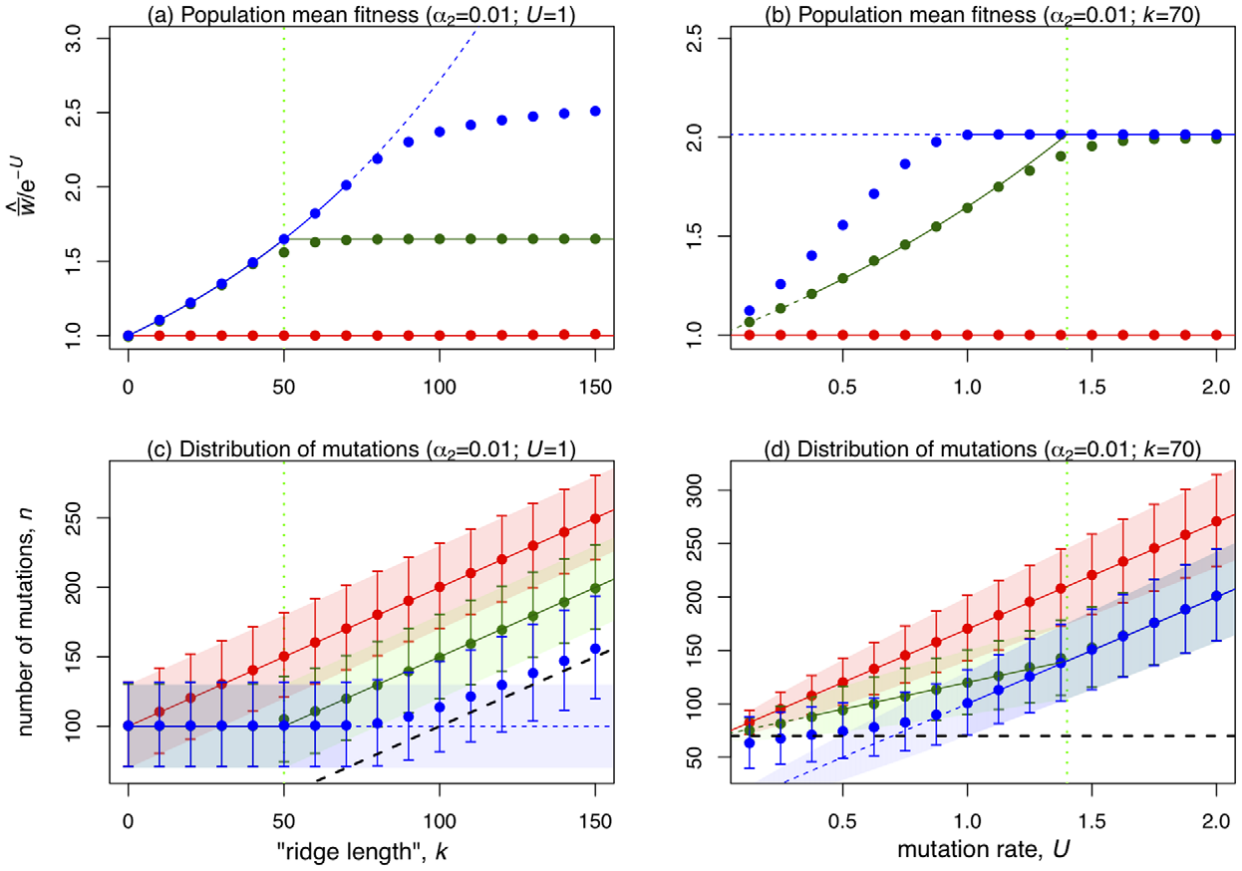
Results for populations at mutation-selection equilibrium in the special case 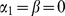. Results for the three modes of reproduction are coloured Red: asexual reproduction; Blue: sexual reproduction with free recombination; and Green: sexual reproduction with segregation alone. The x-axes vary the “ridge length”, 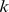 (Panels (a) and (c)), or the genomic deleterious mutation rate, 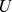 (Panels (b) and (d)). Panels (a) and (b) show the equilibrium mean fitness of the populations, 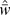. Points show exact numerical results, and solid lines show analytical approximations from Table 1. These solid lines become dashed in regions of parameter space in which hidden epistasis is not predicted (Table 1), and so where the normal approximations might not apply. Panels (c) and (d) represent the genetic constitution of the populations, with points showing the mean number of mutations carried, 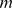, and error bars delimiting 99.75% of the population (corresponding to 3 standard deviations of the normal distribution). Coloured areas show the analytical approximations for 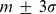 (Table 1). The thick dashed black line shows 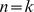, and so populations who do not overlap with this line are said to exhibit hidden epistasis. Dashed coloured lines, and lighter shading denote the parameter regimes where hidden epistasis is not predicted (Table 1). The vertical light-green dotted lines shows the transition between the two possible parameter regimes for sex with segregation alone (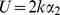).

To see when such hidden epistasis will occur, Table 1 contains analytical approximations and Figure 3 compares these to exact numerical results. Under asexual reproduction, the mean number of mutations at equilibrium is approximately 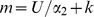, with a variance of 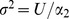 around this mean (Table 1; Figure 3). To derive a stringent condition for hidden epistasis, which will apply even in very large samples, we assume that the distribution of mutations in the population is approximately normal ([19]; see Analysis), and then find the parameters for which 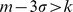, in which case, 99.87% of a normally distributed population will carry 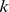 or more mutations. For the type of landscape shown in Figure 2 (i.e., when 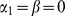), this stringent condition for hidden epistasis will hold for asexuals when 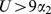 (Table 1).

**Table 1:** Hidden epistasis when 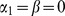.

Reproduction	Regime	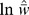	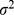	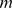
Asexual	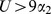	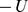	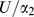	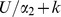
Free recombination	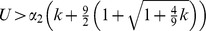	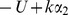	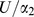	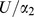
Segregation only	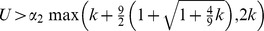	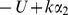	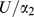	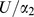
	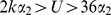	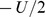	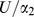	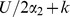

For populations reproducing sexually with free recombination, hidden epistasis can also occur. In the relevant parameter region, sexual individuals carry 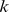 fewer mutations, on average, than asexual individuals with the same parameters (Table 1; Figure 3). This means that the conditions for epistasis being hidden are more stringent, i.e., mutation rates must be higher, but, at equilibrium, sexual mean fitness is larger than asexual mean fitness by the factor 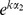. Figure 2b shows an illustrative example, in which sexual populations have a mean fitness about 65% higher than the asexuals, without the epistasis being easily detectable from either population.

Finally, for sexual populations with segregation only, there are two distinct parameter regimes showing hidden epistasis (Table 1; Figures 2 and 3). First, when 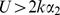, both recombining and non-recombining sexual populations have very similar equilibrium distributions (Figure 2b). However, when mutation rates are lower (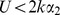), recombination can provide an additional fitness benefit, without epistasis being observable. In this case, a non-recombining sexual population at equilibrium, is fitter than an asexual population by a factor 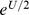, and so the mean fitness benefit due to recombination is 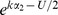 (Figure 2c). Note that this parameter regime only applies when 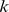 is sufficiently large (from Table 1, no advantage to recombination can occur when 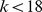, because in this case, 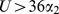, is more difficult to satisfy than 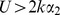).

All of the results above were derived under the assumption of an infinite population, and so it is important to ask when such results will provide insights into real populations of finite size where random genetic drift will operate. The most serious complications introduced by drift apply under asexual reproduction. For example, infinite population results for asexuals will not apply, even approximately, unless the fittest class of individuals is large enough to survive stochastic loss, due to “Muller's ratchet” [5], [8], [17], [20]. Haigh [20] studied the special case of no epistasis (which is 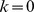 in our notation) and showed that the relative frequency of the fittest class is approximately 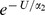. Thus, if 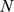 is the size of the (Wright-Fisher) population, then the expected number of individuals in the fittest class at equilibrium is
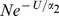
(2)


However, this omits stochastic effects, and so only if 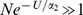, will selection be effective enough to prevent the stochastic loss of this fittest class. If this condition does not hold, then infinite-population results, such as those presented here, will be misleading. In particular, the asexual population will reach an equilibrium in which the number of mutations carried depends almost exclusively on the mutation rates, with little dependence on 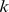. Combining conditions from Table 1 and the results of Haigh [20], we find that hidden epistasis can occur without Muller's ratchet when 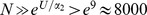. Most natural populations will be large enough for this condition to hold. However, a necessary condition for epistasis to be hidden in freely recombining sexuals is that 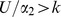 (Table 1). Therefore, absence of the ratchet in asexuals, combined with epistasis remaining hidden in sexuals, requires the far more stringent condition
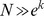
(3)


It is difficult to estimate the biologically relevant range of 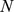, because it lies somewhere between the census population size, and the effective population size, as estimated, for example, from neutral site diversity or fluctuations in allele frequencies. Nevertheless, it is clear that population sizes would have to be very large for asexual populations to gain a fitness advantage over sexuals via hidden epistasis. (When populations are smaller, as is well known, asexuals will still suffer major fitness disadvantages, but for other reasons [5], [8], [17], [20]).

These considerations imply that infinite population results are unlikely to be relevant for asexuals when 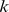 is large. This has two important implications. First, we saw above that a fitness advantage for recombination can only occur when 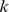 is reasonably large. Together, this imples that hidden epistasis might provide an advantage to either sex or recombination but not, plausibly, for both under any single parameter regime. For example, the (red) asexual curve shown in Figure 2c would be unlikely to hold for any natural population of realistic size. Second, the fitness advantage to sexuals also scales with 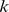 (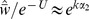), and so, for reasonable values of 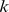, fitness advantages will only be substantial if selection against deleterious mutations is very strong (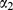 corresponds roughly to the heterozygous selection coefficient against a deleterious mutation).

### Negative versus positive hidden epistasis

**Figure 4:**
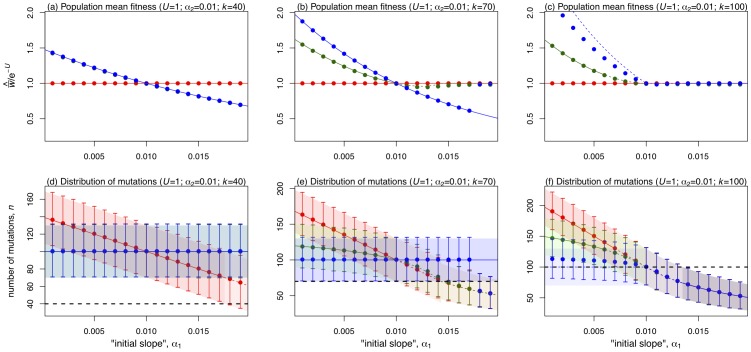
Results for populations at mutation-selection equilibrium in the special case 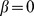. Plots show the equilibrium mean fitness (a)–(c) and the genetic consitution of the population (d)–(f). The x-axes show the decline in fitness for the initial 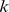 mutations, 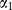. The centre of the plots show results where 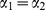, such that no epistasis applies; left of centre (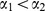) shows negative epistasis, and right of centre (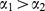) shows positive epistasis. Analytical results were taken from Table 2. All other conventions are as for Figure 3.

In this section, we relax the assumption that the first 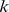 mutations cause no fitness loss, and instead assume that each of these mutations reduces fitness by a factor 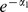. Analytical results for this fitness function are given in Table 2, and compared with exact numerical results in Figure 4. Some of these results are expressed in terms of the compound parameters
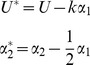
(4)and the results shown will not apply unless both parameters are strictly positive. There are two qualitative changes to the evolutionary outcomes when 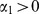. First, there is an additional parameter regime with hidden epistasis, in which almost all individuals carry fewer than 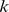 mutations (Figure 4c and 4f). In this case, epistasis can be considered hidden when 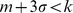, which leads to the condition shown in Table 2. Whenever this inequality applies, the epistasis has no effect, and so all three breeding systems reach the same level of mean fitness, 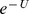 [5].

**Table 2:** Hidden epistasis when 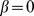.

Reproduction	Regime	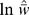	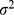	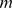
Asexual	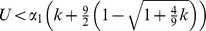	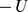	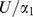	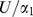
	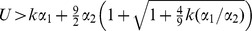	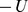	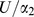	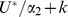
Free recombination	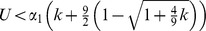	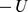	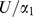	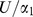
	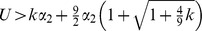	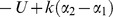	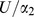	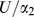
Segregation only	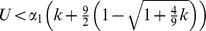	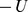	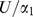	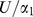
	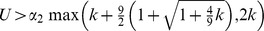	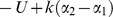	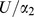	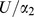
	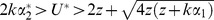 , where 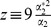	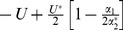	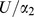	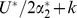

Second, and more interesting, when 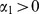 the sign of hidden epistasis can be either negative (when 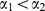) or positive (when 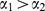). Results with negative epistasis (on the left-hand-side of the plots in Figure 4), do not differ qualitatively from those discussed above. For positive epistasis (the right hand side of the plots in Figure 4), there are cases where sexual reproduction suffers a fitness disadvantage through hidden epistasis (Figure 4a and 4d). However, it is clear from the plots that these regions of parameter space are smaller than the corresponding regions for negative epistasis. For example, in Figure 4c, 4d, 4e and 4f, sexual reproduction with segregation alone can gain a fitness advantage through hidden negative epistasis, but not a disadvantage through hidden positive epistasis; when 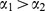, epistasis is either detectable, or all three reproductive systems attain the same mean fitness. This illustrates a point that follows more generally from the inequalities in Table 2: epistasis is less likely to be hidden if it is positive and more likely to be hidden if it is negative. The reason is simple. This kind of hiding of epistasis can occur only if almost all population members have more than 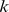 deleterious mutations. Thus, it happens more readily when selection is weak for those individuals that have fewer than 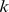 mutations. Negative epistasis means relatively weak selection among individuals with relatively low number of mutations, and this facilitates the hiding of epistasis. This finding may be relevant to interpreting the predominance of positive epistasis from experimental studies with microorganisms [8], [12], [13], [14], [15], [18].

### Negative versus positive hidden epistasis

**Figure 5:**
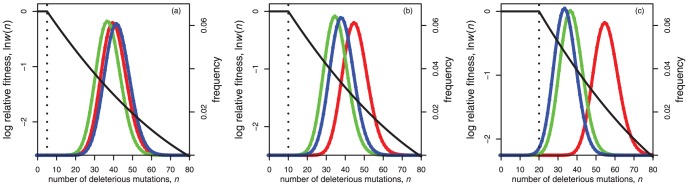
Fitness landscapes and equilibrium frequency distributions. The landscapes shown are identical to that shown in Figure 1c (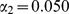, 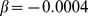) with the exception of an initial neutral ridge (i.e., the first 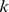 mutations accumulated cause no appreciable loss in fitness). Parameter values are: (a) 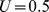, 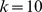, (b) 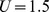, 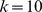; (c) 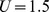, 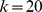. All curves assume an effectively infinite population, and the red (asexual) curve in panel (c) would be unlikely to apply to any population of plausible finite size. All conventions as Figure 1.

Figure 5 shows a fitness landscape that is identical to those shown in Figure 2, except that, for genomes with more than 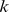 mutations, log fitness is concave upwards (i.e., it exhibits a region of positive epistasis). Parameters in Figure 5 are identical to those used in Figure 1c, with the exception of the neutral ridge (i.e., 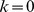 in Figure 1c, and 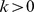 in Figure 5). In all four cases, experimenters would be likely to detect positive epistasis of identical sign and strength (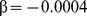 in all cases). However, both hidden and detectable epistasis alter the evolutionary outcomes, and thereby the effects of breeding system on equilibrium mean fitness. For example, the asexual population is fittest in Figure 1c, least fit in Figures 5b and 5c, and of intermediate fitness in Figure 5a.

**Figure 6:**
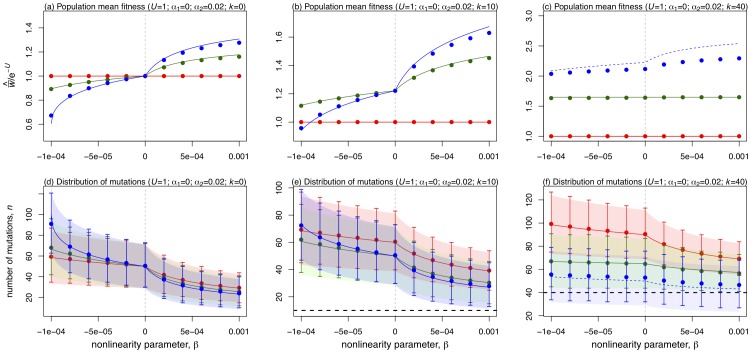
Results for populations at mutation-selection equilibrium. The x-axes show variation in the parameter 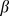, which determines the epistasis following the first 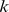 mutations carried. Note that different scales are used for negative and positive values. The analytical results are found in Table 3. Results are shown in the special case 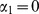, such that the first 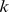 mutations have a negligible effect on fitness (as in Figure 5), and 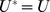, 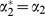 (Eq. 4). All conventions as Figure 3.

Table 3 and Figure 6 contain analytical approximations for this general case. (Results in Table 3 are only shown for mutation rates sufficiently high, and require that the square roots are real numbers, and so when 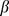 is negative, there is a limited range of validity. However, this restriction is not great, given that 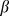 is already constrained by the condition that fitness should not increase with 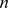 over the relevant range [19]). Figure 6a and 6d show the special case of 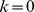, and so replicate the empirically-motivated parameter values chosen by Charlesworth [19].

**Table 3:** Hidden epistasis for a generalized fitness function.

Reproduction	Regime	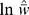	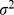	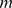
Asexual		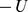	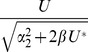	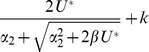
Free recombination		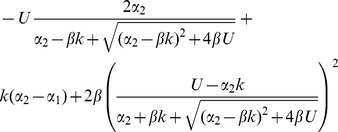	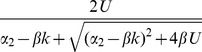	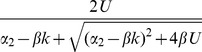
Segregation only	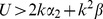	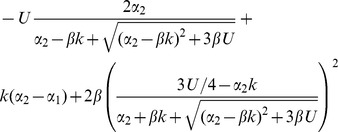	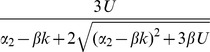	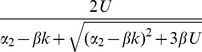
	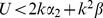	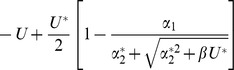	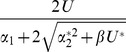	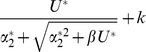

The conclusions and caveats discussed above all apply to these more general results, and in particular, as with Figure 2c, the asexual curve shown in Figure 5c is unlikely to apply to any finite population of plausible size.

## Discussion

We have shown above that the fitness landscape of Eq. (1) as illustrated in Figures 2 and 5, can lead to hidden epistasis. In other words, it can lead to evolutionary outcomes, under different modes of reproduction, that would be not be predicted from the epistasis measured by standard experimental approaches.

Why do fitness landscapes like those shown in Figure 2 have such effects on the relative fitness of sexual and asexual populations? To answer this, note that, when the genomic rate of deleterious mutations, 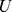, is sufficiently large, the mean number of mutations at equilibrium is virtually independent of 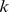 under free recombination, while under asexuality the mean number increases with 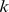 (Table 1). This dependency arises because, under asexuality, the entire population is descended from individuals with relatively small numbers of mutations [23]. Thus, the genetic variance among low-mutation-load individuals affects the genetic variance of the population as a whole. Under asexuality, when 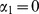, the variance among individuals with 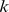 or fewer mutations collapses to zero, because, at equilibrium, all individuals in this category have exactly 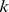 deleterious mutations. (This collapse occurs because, with 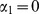, there is no selection between individuals with fewer than 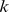 mutations, and this leaves mutation as the only evolutionary force acting among these individuals.) As a result of this collapse, for fitness landscapes such as those shown in Figure 2, the variance in the number of mutations tends to be smaller under asexuality, for a given mean number of mutations, than is the case under free recombination. Thus, under asexuality, more mutations must accumulate than is the case under free recombination before the variance is sufficient to allow selection to remove mutations at the same rate as they are arising (as occurs at equilibrium). This higher mutation load reduces the fitness of asexuals, as compared to a freely-recombining population.

Fitness landscapes that can lead to hidden epistasis might arise through a number of different biological mechanisms [6], [15], [22], [24]–[26]. For example, such landscapes can emerge naturally when mutations affect competitive ability, and when fitness-related competitions tend to occur in small groups [6], [22], [24]. However, it remains to be discovered whether scenarios that can lead to cryptic negative epistasis are common in nature. A direct (but difficult) way to find out would be to generate (or identify) individuals that have many fewer deleterious mutations than the typical member of a natural population, and then measure their fitness under realistic circumstances. Other possible approaches depend on the mechanism responsible for epistatic interactions. For example, if epistasis arises from competition [6], [22], [24], then a practical approach might be to compete wild-type individuals against members of a population that has been debilitated in some way or other, such as by rearing with inadequate supplies of nutrients [27]. The aim is to see whether a ceiling effect arises, such that individuals that are exceptionally vigorous (in comparison to the debilitated population) cannot gain much more fitness by becoming even more vigorous. A similar approach would be to use populations derived from mutation-accumulation studies [28], [29].

There are several important caveats to the results presented here. First, our results concern equilibrium mean fitness, which can play an important role in the evolution of sex [30]. However, equilibrium mean fitness may not be predictive of the fate of modifiers of recombination, which previous results suggest will depend on the local epistasis, detectable by standard experimental approaches [1], [19]. Second, our results for asexuals depend on the very fittest individuals in the population being present in substantial numbers [6], [19]–[21], while, by definition, hidden epistasis requires that such individuals form a negligible proportion of the total population (or at least be absent from typical experimental samples). This implies that our results for asexuals, like other similar treatments, will apply only when population sizes are very large, and also limits the size of the fitness benefit that hidden epistasis can provide (since larger benefits require ever larger populations). It also implies that there is no region of parameter space in which both sexual segregation and recombination might plausibly yield mean fitness advantages through hidden epistasis. Finally, the exact size of the advantage gained through hidden epistasis is certain to depend on the complications of real-world fitness landscapes, and on genetic details that have been simplified here [8], [17], [31].

## Analysis

### Normal approximation

We begin with approximate results that apply regardless of the mode of reproduction, and which will be used repeatedly below. Let 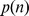 denote the probability that a randomly picked individual in the population carries 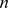 mutations; this is the distribution of the number of mutations per individual. We then follow Charlesworth [18], [19] by approximating the discrete distribution at equilibrium, 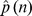, by a continuously-valued normal distribution:
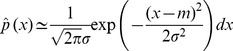
(5)


This approximation can be used to obtain an expression involving the variance in the number of mutations at equilibrium, 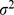. To derive this approximation, we note that at equilibrium, regardless of the mode of reproduction, the number of mutations after selection and mutation, will be equal to 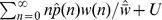. We therefore have:
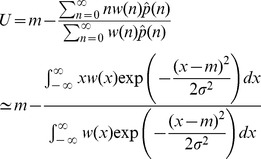
(6)


Using the identity
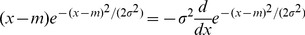
(7)and integrating by parts yields
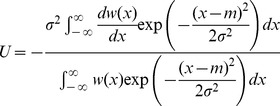
(8)


We then make a further approximation by neglecting deviations of 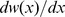 from its value at 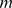, to obtain
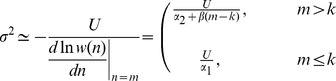
(9)


We can make a similar approximation for the population mean fitness at equilibrium, assuming normality, and then approximating 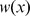 by its value evaluated at 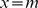 (i.e., neglecting the deviation of 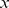 from 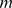 within 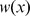). This yields:
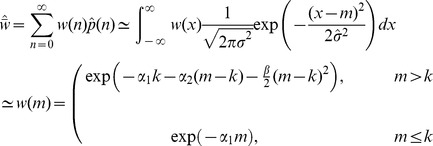
(10)


Together, Equations (5), (9) and (10) provide all of the results we require, with the exception of the mean number of mutations at equilibrium, 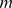, for each mode of the reproduction. In the following sections, we derive the results for 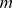.

### Asexual reproduction

Under asexual reproduction, offspring are genetically identical to their parent, apart from new mutations. In this case, the distribution of the number of mutations per individual evolves according to
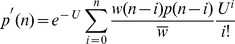
(11)(Eq. 3 of [5]). Iterating this equation provides the exact numerical results for asexual reproduction shown in Figures 1, 2, 3, 4, 5, and 6. Kimura and Maruyama [5] showed that the equilibrium mean relative fitness in this case is given by
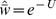
(12)regardless of the precise form of the fitness function. Substituting in Eq. (12) to Eq. (10) yields 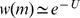. Assuming 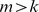, then yields a quadratic equation in 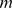. Of the two possible roots, only one leads to a positive value of 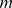 for both 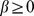 and a finite range of negative 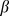. Numerically, we find that this solution, shown in Tables 1, 2, and 3, applies throughout the range of parameters considered here. The same procedure, but assuming 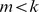, gives the non-epistatic equilibrium found in Table 2. Using these results in Eqs. (9) and (10) provide the results for 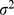 and 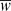 in the tables, and these can used to derive our conditions for hidden epistasis, which are equivalent to 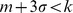 or 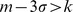.

### Sexual reproduction with free recombination

To model sexual reproduction, we consider a population of diploid hermaphrodites, whose offspring are produced by the random union of haploid gametes. If we assume free recombination between all sites, then the distribution of the number of mutations per individual evolves according to:
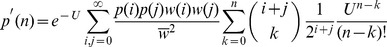
(13)
[5]. This recursion was used to derive the exact results for freely recombining populations in Figures 1, 2, 3, 4, 5, and 6. To make analytical progress, we note that with free recombination, the variance in the number of mutations carried at equilibrium is approximately equal to its mean, i.e., 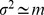 [19]. We therefore substitute 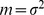 directly into Eq. (9), which yields a quadratic in either 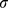 or 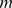. Other results in Tables 1, 2, and 3 then follow as described above for the asexuals.

### Sexual reproduction with segregation only

For sexual reproduction without recombination, we again assume diploid hermaphrodites and the random union of gametes. But now, each gamete is assumed to consist of a single chromosome, which is produced without recombination at meiosis. In this case, it is easier to consider the dynamical equation for the number of mutations per haploid gamete [5]. As such, we denote as 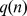 the probability that a randomly chosen gamete carries 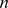 deleterious mutations. The dynamical equation for 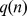 is
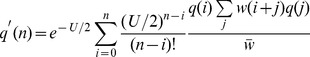
(14)

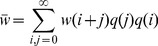
(15)
[5]. Considering Eqs. (14)–(15) at equilibrium, setting 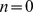, and assuming 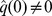, yields
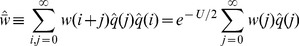
(16)


We then apply Charlesworth's [19] normal approximation, Eq. (5), directly to the distribution of gametes, i.e., we approximate 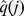 by a normal distribution with mean 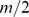 and variance 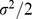. This yields
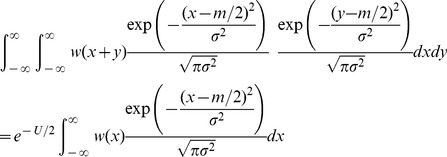
(17)We now approximate further by neglecting deviations in 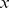 and 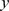 from their mean values within 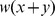, to obtain
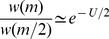
(18)


Equation (18) has three possible solutions for 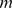, depending on the region of the fitness function occupied by the population. If 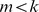, we obtain the non-epistatic equilibrium results given in Table 2. If 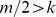 we obtain the higher mutation rate result, which corresponds to the outcome with free recombination when 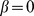 (Tables 1 and 2). Finally, when 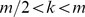 (such that zygotes, but not gametes, carry more than 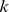 mutations), there is a distinct outcome where recombination can further increase mean fitness. Solving these inequalities for 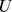 provides the additional conditions for these outcomes shown in Tables 1, 2, and 3.
